# Implication of human papillomavirus-66 in vulvar carcinoma: a case report

**DOI:** 10.1186/1752-1947-5-232

**Published:** 2011-06-25

**Authors:** Ioannis C Kotsopoulos, Georgios P Tampakoudis, Dimitrios G Evaggelinos, Anastasia I Nikolaidou, Panagiota A Fytili, Vasilios C Kartsiounis, Domniki K Gerasimidou

**Affiliations:** 1 Gynecological Oncology Department, "Theagenio" Cancer Hospital, 2 Alex. Simeonidis Street, Thessaloniki, 54007, Greece; 2 Pathology Department, "Theagenio" Cancer Hospital, 2 Alex. Simeonidis Street, Thessaloniki, 54007, Greece; 3 Cytology Department, "Theagenio" Cancer Hospital, 2 Alex. Simeonidis Street, Thessaloniki, 54007, Greece

## Abstract

**Introduction:**

Vulvar cancer in older women is seldom associated with human papillomavirus infection.

**Case presentation:**

We present the case of an 80-year-old Greek Caucasian woman with an undetermined obstetric and gynecologic history. The patient underwent radical vulvectomy and bilateral inguinal lymphadenectomy for a vulvar carcinoma. A human papillomavirus infection was suggested on the basis of histological and cytological examinations followed by human papillomavirus DNA typing, which revealed the presence of human papillomavirus-66.

**Conclusion:**

Even though human papillomavirus-16 and human papillomavirus-18 are most frequently implicated in the pathogenesis of vulvar carcinoma, human papillomavirus-66 can also be regarded as a causative factor. Suspicious lesions should be biopsied, and in the presence of carcinoma, vulvectomy with bilateral lymphadenectomy, if necessary, must be performed. Furthermore, polymerase chain reaction assay analysis with clinical arrays in cytological samples is an accurate test for the detection of a wide range of human papillomavirus genotypes and can be used to verify the infection and specify the human papillomavirus type implicated.

## Introduction

Vulvar carcinoma in older women is seldom associated with any type of human papillomavirus (HPV) infection, representing less than 15% of reported cases [1]. Atypia of the squamous epithelium of the vulva is considered to be a co-factor in the carcinogenesis of vulvar cancer and usually a non-neoplastic lesion, such as vulvar inflammation, lichen sclerosus or hyperplasia of squamous cells, pre-exists [1]. Two models have been suggested for the development of vulvar cancer [1]. Type 1 occurs in relatively young women and is associated with warty or basaloid vulvar intra-epithelial neoplasia. According to its definition, type 2 is represented by keratinizing squamous cell carcinoma and mainly presents in post-menopausal women, and its association with HPV infection is quite rare (< 15%). Although smoking and sexually transmitted diseases are considered to be co-factors in type 1 vulvar cancer, there is a low correlation with type 2 [1]. The most common HPV genotypes in vulvar carcinoma are types 16 and 18, while genotypes 31, 33, 45, 52, 53 and 62 have also been considered as etiological factors [2-5]. HPV-66 is a rare type of α-papillomavirus. Even though the prevalence and distribution of HPV-66 in most studies have depended highly upon the origin of the population involved, in a meta-analysis of carcinomas and intra-epithelial neoplasia of the vulva, vagina and anus, HPV-66, among other rare types, was found in no more than 0.5% of any anogenital carcinomas that were tested [6]. This type has also been associated with cervical intra-epithelial neoplasia 1 lesions and *Verruca vulgaris *[7,8]. On the basis of a review of the latest international literature, we found only one other case in which the co-existence of HPV-66 and vulvar carcinoma was reported; however, it was reported in combination with HPV-52 infection [9].

## Case presentation

In this report, we describe the case of an 80-year-old woman of Greek Caucasian origin, gravida 2 para 2, with an undetermined obstetric and gynecologic history. After her second delivery, no data referring to the clinical history of the patient was available because the patient deferred any preventive or diagnostic medical examination until she presented to our hospital. Her last menstrual period had occurred 28 years ago. Her medical history included hypertension and angina pectoris.

**Figure 1:**
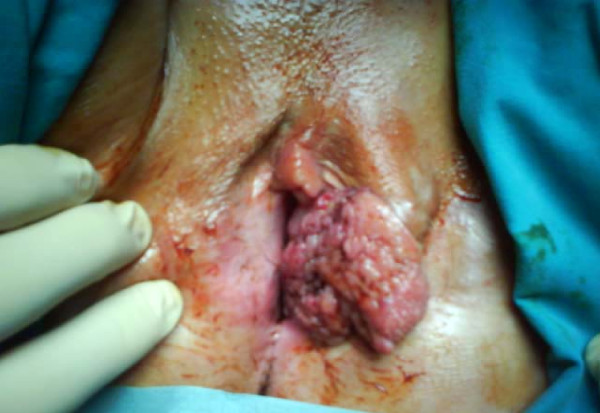
**Preoperative image of the lesion**. Α 4 cm × 5 cm warty lesion is present on the left labium majus of the vulva.

The patient was examined in the gynecological oncology outpatient department and was found to have chronic pruritus vulvae and a recent onset palpable inflammatory lesion on the left labium majus. The lesion bled occasionally. During her gynecological examination, a 4 cm × 5 cm warty lesion with ulcerous and hemorrhagic areas (Figure 1) was found in the middle of the left labium majus.

Initially, multiple biopsies from the center of the lesion and from the lateral margins were obtained, the histological examination of which revealed the presence of squamous cell carcinoma. At the periphery of the lesion, the squamous stratified epithelium exhibited abnormalities consistent with vulvar intra-epithelial neoplasia (vulvar intra-epithelial neoplasia (VIN) grade I/II). Numerous lesional cells showed koilocytic atypia, which is representative of HPV-related infection. However, initial hybrid capture 2 testing for HPV was negative in all the samples tested, which were obtained from either the center or the periphery of the lesion. A pre-operative computed tomographic scan of the abdomen and inguinal areas showed bilaterally enlarged inguinal lymph nodes with central fusion.

The patient underwent radical vulvectomy extending centrally to the level of the perineal membrane, as well as bilateral inguinal lymphadenectomy. Post-operatively, the woman had no major complications and was discharged 14 days after the procedure.

**Figure 2:**
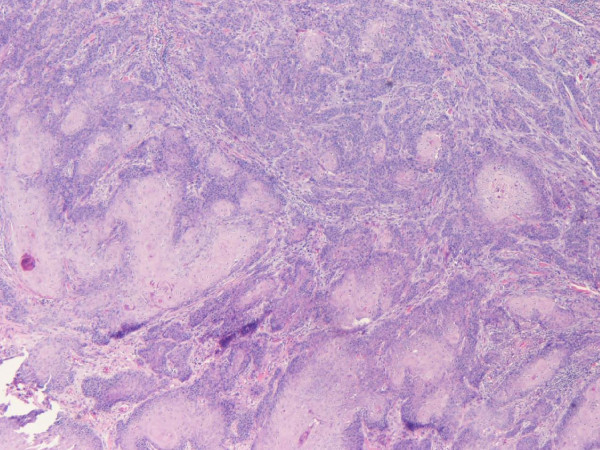
**Moderately differentiated architectural and cytologic appearance of squamous cell carcinoma among mildly desmoplastic stroma (hematoxylin and eosin stain; original magnification, ×100)**.

**Figure 3:**
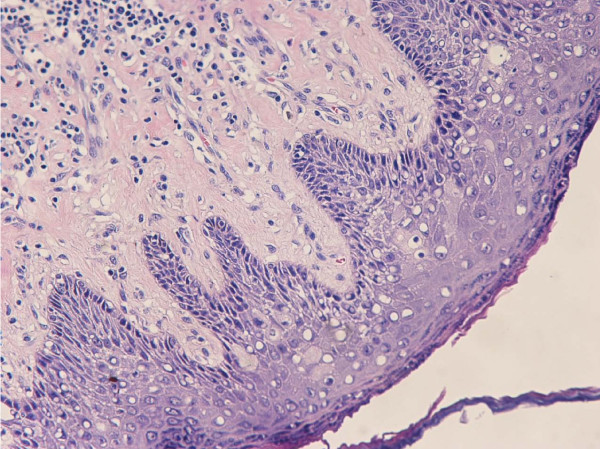
**Koilocytic changes in vulvar squamous epithelium consistent with HPV infection (hematoxylin and eosin stain; original magnification, ×200)**.

**Figure 4:**
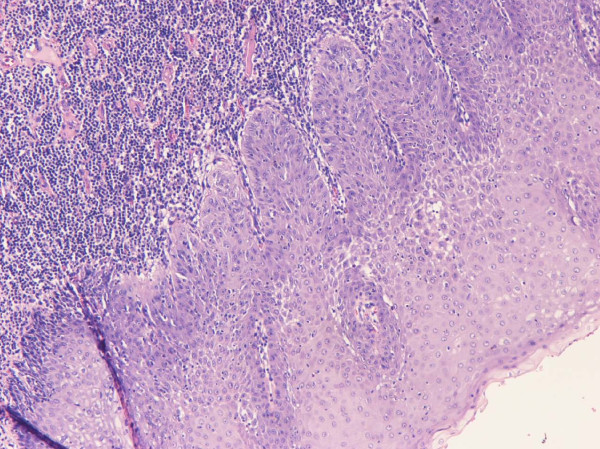
**Vulvar intra-epithelial neoplasia (VIN grade I) adjacent to carcinoma (hematoxylin and eosin stain; original magnification, ×100)**. There is proliferation and atypia of the lower third, but surface maturation is evident. The stroma is heavily infiltrated.

A histopathological examination of the excised specimen verified the presence of squamous cell carcinoma grade II/III with superficial ulcerations (Figures 2 and 3). Carcinoma cells invaded the stroma and the underlying adipose tissue with irregular invasive margins. The full thickness of the lesion from the surface to the deepest point was 1.2 cm. As noted regarding the pre-operative biopsies, the adjacent squamous stratified epithelium exhibited VIN grades I and II lesions (Figure 4). Furthermore, metastases of the squamous cell carcinoma involving two of 11 right inguinal lymph nodes and two of five left inguinal lymph nodes were found. The vulvar lesion was excised within normal tissues.

In the tissue specimen obtained pre-operatively for the performance of liquid-based Cytology (The Thin prep - pap test, Cytyc Corp., Marborough, Massachusetts, USA), we used a polymerase chain reaction (PCR) assay with CLINICAL ARRAYS Human Papillomavirus Kit (Genomica, Madrid, Spain) and a Hybrid Capture 2 HPV DNA test (Digene, Madrid, Spain). The latter test is an accurate qualitative and quantitative method used to detect five low-risk types of HPV (6, 11, 42, 43 and 44) and 13 high-risk types of HPV (16, 18, 31, 33, 35, 39, 45, 51, 52, 56, 58, 59 and 68). It can also determine the viral load. This technology is based on hybrid analysis to identify indentifying a signal enhancement in a microplate using chemiluminescence. Samples containing the target DNA are hybridized with a specific detector HPV RNA. Hybrid-produced RNA-DNA binds to the surface of a microplate coated with specific antibodies for RNA-DNA hybrids. The immobilized hybrids react with alkaline phosphatase (ALK)-conjugated antibodies and are detected with a chemiluminescence substrate. While the binding ALK cleaves the substrate, it attracts light, which is measured by chemiluminescence in relative light units [10].

**Figure 5:**
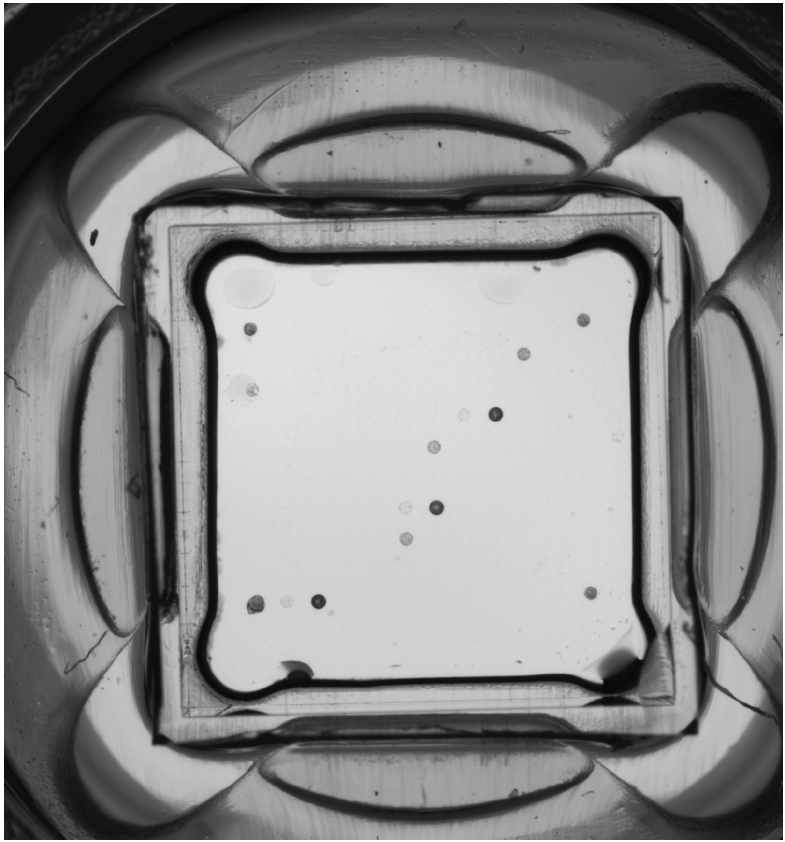
**The CLINICAL ARRAYS Human Papillomavirus Kit was used for HPV typing**. The combination of the three dark diagonal points indicates the presence of HPV-66 typing. The other, less prominent dots represent control markers.

For the purpose of this study, we also used the CLINICAL ARRAYS Human Papillomavirus Kit (Genomica, Madrid, Spain), which is a commercially available HPV genotyping microarray test (Figure 5). The kit was used according to the manufacturer's protocol. We used 10 μL of purified DNA for each specimen. The kit employs biotinylated primers to define a sequence of 451 nucleotides within the polymorphic L1 region of the HPV genome. A pool of HPV primers is used to amplify HPV DNA from 35 mucosal genotypes, including 15 high-risk genotypes (16, 18, 31, 33, 35, 39, 45, 51, 52, 56, 58, 59, 68, 73 and 82), three potentially high-risk genotypes (26, 53 and 66), 11 low-risk genotypes (6, 11, 40, 42, 43, 44, 54, 61, 70, 72 and 81) and six genotypes of unknown risk (62, 71, 83, 84, 85 and 89). In addition to the microarray analysis, PCR was performed with the HPV consensus primers MY09 and MY11 (18). PCR was performed consecutively as follows: 95°C for 15 minutes and 40 cycles of 94°C for 15 seconds, 52°C for 30 seconds and 72°C for 45 seconds [11].

In our case, none of the above-mentioned HPV types was detected with the use of hybrid capture 2 testing. The DNA of HPV-66 was the only one detected using the CLINICAL ARRAYS Human Papillomavirus Kit.

On the basis of these results, a standard PCR assay was also performed. Four histological specimens from four different levels of the lesion, as well as from a lymph node with metastasis, were examined. Extraction of total DNA from formalin fixed, paraffin-embedded sections was performed using the QIAamp DNA Mini Kit (Qiagen, Hilden, Germany) according to the manufacturer's instructions. The quality and integrity of extracted DNA were assessed by PCR amplification of an amplicon of the interferon (IFN)-γ gene and by agarose gel (0.6%) electrophoresis, respectively. A nested, multiplex, highly sensitive PCR method (approximately 1 fg/103 viral copies) was used for HPV detection and genotyping. In this assay, consensus primers for first-run amplification of a broad spectrum of mucosal HPV genotypes, including all high-risk HPV genotypes, were combined with nested PCR amplifications of type-specific primers. Despite robust detection of the IFN-γ gene product amplification, no positive signal for HPV presence was found in any of the examined samples, even after substantially increasing the PCR cell cycles. Considering that there were clear indications of HPV infection in the microscopic examination of the specimens, the PCR assay was unable to detect the DNA of the virus, most probably because of low viral load in the specimens examined, which could have been caused and amplified by inadequate superficial lesional tissue. Furthermore, tissue fixation techniques could have a negative impact on the preservation of the viral genome.

## Discussion

The presence, coexistence and possible cause of HPV infection in women's anogenital squamous neoplasia have been studied extensively over the past decade. It must be emphasized that the presence of condylomatous lesions does not exclude the possibility of a coexisting invasive malignancy. However, the correlation appears to be stronger as far as intra-epithelial lesions (VIN grade III) are concerned.

HPV-66 is an α-papillomavirus considered to belong among the potentially high-risk mucosal HPV types. Nevertheless, it can also be encountered in benign lesions. Even though this HPV type is reported to be associated with cervical squamous carcinomas, little is known concerning vulvar squamous cell carcinomas. On the other hand, vulvar carcinomas are mainly correlated with HPV-16 and 18 genotypes. In our case report, we describe the case of a woman with a HPV-66-related vulvar carcinoma. This diagnosis was made on the basis of the use of PCR with the CLINICAL ARRAYS Human Papillomavirus Kit.

## Conclusion

In conclusion, in patients with HPV-66 infection the possibility of a coexisting invasive malignancy, albeit rare, should be considered even in the presence of benign lesions. Caution should be taken, especially in older women with cancer, as the majority of these cancers are HPV-negative. In cases that raise clinical suspicions of HPV, examination of tissue using PCR with the CLINICAL ARRAYS Human Papillomavirus Kit should be considered in patients with histological features of HPV infection and negative Hybrid Capture 2 assay testing, as it detects a wider range of HPV genotypes than other types of testing, including HPV-66. A standard PCR assay of formalin-fixed samples seemed to be less effective, as it appeared to be affected by sampling, tissue fixation and/or viral load. Patients should be followed up meticulously at short time intervals.

## Consent

Written informed consent was obtained from the patient for publication of this case report and accompanying images. A copy of the written consent is available for review by the Editor-in-Chief of this journal.

## Competing interests

The authors declare that they have no competing interests.

## Authors' contributions

IK conceptualized the case report, collected and analyzed all data and wrote the major part of the manuscript. GT corrected the initial manuscript and wrote parts of the manuscript. DE was the major gynecologist (in cooperation with IK and GT) who cared for and conducted patient follow-up. DG and AN performed the histological examinations and corrected the pathological parts of the manuscript. Also, DG wrote parts of the manuscript and gave final approval of the manuscript. PF performed the HPV typing (Hybrid Capture 2 assay and CLINICAL ARRAYS Human Papillomavirus Kit testing) and corrected the cytological parts of the manuscript. VK reviewed the literature and wrote some parts of the Introduction. All authors read and approved the final manuscript.
